# Contrasting Genetic Structure in Two Co-Distributed Species of Old World Fruit Bat

**DOI:** 10.1371/journal.pone.0013903

**Published:** 2010-11-10

**Authors:** Jinping Chen, Stephen J. Rossiter, Jonathan R. Flanders, Yanhong Sun, Panyu Hua, Cassandra Miller-Butterworth, Xusheng Liu, Koilmani E. Rajan, Shuyi Zhang

**Affiliations:** 1 Guangdong Entomological Institute, Guangzhou, China; 2 School of Biological and Chemical Sciences, Queen Mary University of London, London, United Kingdom; 3 School of Biological Sciences, University of Bristol, Bristol, United Kingdom; 4 Huazhong Normal University, Wuhan, China; 5 School of Life Sciences, East China Normal University, Shanghai, China; 6 Department of Human Genetics, University of Pittsburgh, Pittsburgh, Pennsylvania, United States of America; 7 Department of Animal Science, School of Life Sciences, Bharathidasan University, Tamil Nadu, India; University of Poitiers, France

## Abstract

The fulvous fruit bat (*Rousettus leschenaulti*) and the greater short-nosed fruit bat (*Cynopterus sphinx*) are two abundant and widely co-distributed Old World fruit bats in Southeast and East Asia. The former species forms large colonies in caves while the latter roots in small groups in trees. To test whether these differences in social organization and roosting ecology are associated with contrasting patterns of gene flow, we used mtDNA and nuclear loci to characterize population genetic subdivision and phylogeographic histories in both species sampled from China, Vietnam and India. Our analyses from *R. leschenaulti* using both types of marker revealed little evidence of genetic structure across the study region. On the other hand, *C. sphinx* showed significant genetic mtDNA differentiation between the samples from India compared with China and Vietnam, as well as greater structuring of microsatellite genotypes within China. Demographic analyses indicated signatures of past rapid population expansion in both taxa, with more recent demographic growth in *C. sphinx*. Therefore, the relative genetic homogeneity in *R. leschenaulti* is unlikely to reflect past events. Instead we suggest that the absence of substructure in *R. leschenaulti* is a consequence of higher levels of gene flow among colonies, and that greater vagility in this species is an adaptation associated with cave roosting.

## Introduction

Comparative studies taxa offer a powerful approach to elucidate how population processes and historical events have acted in shaping organisms' current distribution and genetic structure. Spatially matched taxa are often expected to show similar signatures of structure based on their shared histories [1]. For example, several studies have reported concordant patterns of genetic structure in Europe, increasing our understanding of the impact of current and past barriers to gene flow [2], [3], [4], [5]. Conversely, disparities in genetic structure between co-distributed species can highlight differential responses to these processes often resulting from contrasting life history and ecological traits.

Dispersal ability is a key demographic force shaping natural populations [6]. Contrasting dispersal strategies, which are often linked with social structure, impact on the balance between gene flow, genetic drift, mutation and natural selection [7]. In general, species with limited dispersal abilities show more population genetic structure than do species with a tendency towards greater dispersal [8]. In bats, social structure is largely determined by roosting ecology. Natural caves provide one type of roosting habitat, but their patchy distribution in the landscape and potential to accommodate large numbers of individuals means that the number of bats using one cave can range from hundreds to thousands. Consequently, competition for local resources is expected to be high and many cave roosting bat species typically exhibit high vagility, thus allowing them to commute large distances to foraging sites [9]. Conversely, tree cavity/foliage roosting species of bats tend to live in much smaller numbers in a ubiquitous resource. This might have led to less evolutionary pressures for high dispersal. These expectations are supported by empirical data that show cavity/foliage roosting species are more susceptible to fragmentation than cave-roosting species [10].

The fulvous fruit bat (*Rousettus leschenaulti*) and the greater short-nosed fruit bat (*Cynopterus sphinx*) are co-distributed throughout much of their range in Southern Asia [11], [12]. Although both species are frugivorous they exhibit markedly different roosting behavior with *R. leschenaulti* predominantly roosting in caves consisting of mixed sex colonies that can consist of up to 10,000 individuals [13]. In contrast, *C. sphinx* typically roosts in palm trees during the breeding season, and is characterized by a single male with a harem of females [7].

By occupying a similar range but having different roosting preferences, *R. leschenaulti* and *C. sphinx* provide us with an opportunity to test whether expected differences in dispersal show a correlation to population genetic structure. In this study, we aim to compare the population genetic structure of *R. leschenaulti* and *C. sphinx* over a wide geographical area using both mitochondrial (cytochrome *b* gene) and microsatellite markers. We predict that *R. leschenaulti* will have a high dispersal ability that will be characterized by the identification of low population genetic structure across its range, while the opposite will be found for *C. sphinx* due to its predicted limited dispersal ability.

## Materials and Methods

### Ethics statement

Sampling was approved by the Administrative Panel on Laboratory Animal Care (approval number 2009001) of the Guangdong Entomological Institute in China, which also incorporates the South China Institute of Endangered Animals.

### Sample collection

**Figure 1:**
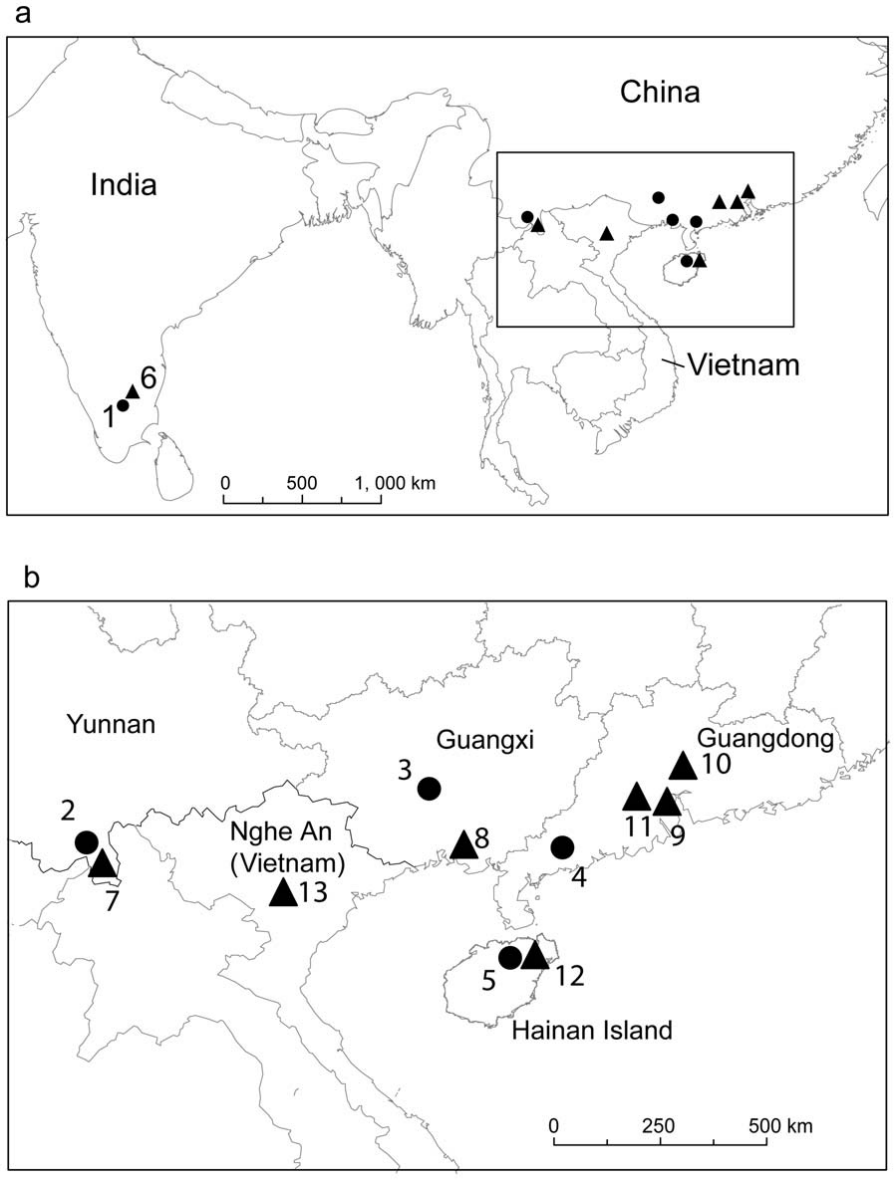
Sampling locations for *Cynopterus sphinx* and *Rousettus leschenaulti.* Map of the sampling locations for *Cynopterus sphinx* (triangles) and *Rousettus leschenaulti* (circles) across a) whole sampling range and b) detailed view of sampling sites and provinces in China and Vietnam.

*Rousettus leschenaulti* was sampled at four localities in China (Maoming, Wuming, Haikou, Menglun) and one in India (Cheranmahadevi). *Cynopterus sphinx* was sampled at six localities in China (Guangzhou, Zhongshan, Jiangmen, Beihai, Haikou, Xishuangbanna), one in India (Mandiyoor) and one in Vietnam (Pu Huong) (Table 1, Figure 1). To avoid over-representation of potential relatives, bats of both species were captured using mist nets at foraging grounds (or for *Rousettus*, also near to roost entrances) rather than by hand-netting. Tissue samples were taken from the wing-membrane using a 3-mm diameter biopsy punch and stored in 80% ethanol until processing [5]. All bats were released at the site of capture

**Table 1:** Details of sampling locations in a) five populations of *Rousettus leschenaulti* and b) eight populations of *Cynopterus sphinx*.

	Location	Locality	Province	Country	Easting	Northing	n
a)	1	Cheranmahadevi	Tamil Nadu	India	E77:42	N8:44	20
	2	Menglun	Yunnan	China	E101:15	N21:55	25
	3	Wuming	Guangxi	China	E108:17	N20:03	32
	4	Maoming	Guangdong	China	E110:53	N21:40	39
	5	Haikou	Hainan Island	China	E110:20	N20:02	41
b)	6	Mandiyoor	Tamil Nadu	India	E78:43	N10:55	19
	7	Xishuangbanna	Yunnan	China	E101:25	N21:41	31
	8	Beihai	Guangxi	China	E109:07	N21:28	22
	9	Jiangmen	Guangdong	China	E113:04	N22:35	14
	10	Guangzhou	Guangdong	China	E113:23	N23:09	64
	11	Zhongshan	Guangdong	China	E113:22	N22:32	18
	12	Haikou	Hainan Island	China	E110:20	N20:02	36
	13	Pu Hong	Nghe An	Vietnam	E105:15	N20:22	14

Locality, province, country, geographic co-ordinates (Easting and Northing), and sample size (n) are shown.

### DNA extraction and microsatellite genotyping

DNA was extracted using DNeasy Tissue kits (Qiagen). *R. leschenaulti* was genotyped at eight microsatellite loci while *C. sphinx* was genotyped at six (see Table S1). To amplify microsatellite loci, polymerase chain reactions (PCRs) were undertaken using total reaction volumes of 10 µl, each containing 50–100 ng genomic DNA, 0.25 µM of each primer, and 1x PCR buffer containing 2 mM MgCl_2_, 0.2 mM of each dNTP, and 0.25 U hot-start Taq DNA polymerase (Qiagen). Forward primers were 5′-fluoro-labelled (MedProbe). PCRs were performed using a PTC-200 thermal cycler (MJ Research) with the following profile: denaturation at 95°C for 15 min, 35 amplification cycles (94°C for 30 s, annealing temperature for 30 s, 72°C for 30 s) and an extension step at 72°C for 20 min. PCR products were genotyped using an ABI 3100 DNA sequencer (Applied Biosystems) and alleles were sized using the programs GENESCAN version 2.1 and GENOTYPER version 2.5 (Applied Biosystems).

### mtDNA sequencing

Five to eight individuals from each population (total n = 88) were amplified at the cytochrome *b* gene (∼1100 bp) using the published primers cy1 (5′-AAA TCA CCG TTG TAC TTC AAC-3′) and cy2 (5′-TAG AAT ATC AGC TTT GGG TG-3′) [14]. PCRs were performed using a PTC-220 thermal cycler (MJ Research) in 50 µl reaction volumes, each containing 25 µl of 2× ExTaq polymerase (Takara), 0.5 µl of DNA templates (50 µg/µl), and 2 µl of each primer (10 µM). For PCR conditions see the microsatellite genotyping section above. PCR products were sequenced using Big Dye Terminator kits (Applied Biosystems) on an ABI 3730 automated sequencer with both primers.

### Microsatellite statistical analyses

We tested for deviations from linkage equilibrium between loci in FSTAT version 2.9.3.2 [15], and from Hardy-Weinberg equilibrium (HWE) for each population and locus using the Markov chain method (10,000 dememorization steps, 10,000 batches and 10,000 iterations per batch) in GENEPOP version 3.3 [16]. Population values of the mean number of alleles per locus and heterozygosity (observed and expected) was calculated using GENEPOP. We also estimated allelic richness (R_S_) per locus per population, which accounts for unequal sample sizes, in FSTAT. To estimate the power of the markers, we calculated the total probability of identity’ (PID) for each locus and for all loci combined in GENALEX [17].

To quantify genetic structure, we calculated pairwise F_ST_ values using the software GENETIX version 4.02 [18]. To test for a phylogeographic signal in the data, we also derived pairwise R_ST_ values, which accounts for the stepwise mutation model of microsatellite alleles [19], and compared these to R_ST_ values in which allele sizes were permuted (1000 times) among alleles (_p_R_ST_) [20] using the software SPAGeDi version 1.3 [21]. The extent to which R_ST_ exceeds _p_R_ST_ is thus a measure of the extent to which phylogenetic distance among ordered alleles explains the observed pattern of genetic differentiation. We then assessed the relative contribution of phylogeography between the two species by using a paired sample t-test on equivalent pairwise values of R_ST_ - _p_R_ST_.

We also plotted linearized pairwise values of both F_ST_ and R_ST_ against log-transformed geographic distances (km) to test for isolation by distance (IBD), using a Mantel test with permutations (1000 times) to assess significance in GENEPOP. An analysis of molecular variance (AMOVA) was used to examine genetic variation within and among the different populations, based on all samples, with and without the Indian colony (AMOVA I and II, respectively). We assessed whether the derived indices were significantly different from zero using a permutation procedure (5,000 iterations) in the software ARLEQUIN version 3.1 [22].

Population structure was further investigated using Bayesian clustering analysis. We estimated the likelihood of different numbers of clusters (K) in the data using the method in STRUCTURE version 2.0 [23]. Five independent runs (burn-in of one million and one million MCMC steps), were undertaken for each value of K from 1 to 8. We applied the admixture symmetric similarity coefficients (SSC) among replicate runs within each value of K [24], using the Greedy algorithm of CLUMPP version 1.1.1 [25] in which groups of runs with a SSC≥0.8 were identified and combined. Summary outputs for each value of K were then displayed graphically using the software DISTRUCT version 1.1 [26].

### Sequence analysis

Cytochrome *b* sequences were aligned in BioEdit version 7.0.5 [27] and both haplotype diversity (h) and nucleotide diversity (π) were calculated for each population in the software DNASP version 4.0 [28]. For each species, the relationship among haplotypes was assessed using a network method based on statistical parsimony. Haplotype networks were constructed in the program TCS version 1.21 [29] with the maximum number of mutational differences justified by the parsimony limit of 0.95.

Tests for genetic differentiation among samples (n≥5), calculated using pairwise Φ_ST_ values and tested for significance by permutation (10 000). AMOVAs were used to examine genetic variation following the methods described above. Finally, to test whether any detected differences in population structure between the two species could be explained by contrasting demographic histories, we performed sequence mismatch distribution analyses in ARLEQUIN. Mismatch distributions are typically ragged or multimodal for populations at stationary demographic equilibrium, but smooth or unimodal for populations that have undergone a demographic expansion [30]. Goodness of fit tests for a model of population expansion were calculated from the sum of squared deviation (SSD) and the raggedness index (r), and significance was assessed by bootstrapping (10 000 replicates). Where evidence of population expansion was found, the expansion time in generations (t) was derived following t = T/2*u*, where Τ (tau) is a parameter of the time to expansion in units of mutations, and *u* is the mutation rate per generation for the DNA sequence. We used a mutation rate of 2% per Myr [31] with a generation time of 2 years, based on age of first breeding for most insectivorous bat species [32].

## Results

### Genetic diversity

Samples of both *Rousettus leschenaulti* and *Cynopterus sphinx* showed no evidence of linkage disequilibrium between loci, and there was no significant deviation from Hardy-Weinberg equilibrium detected in either species following Bonferroni correction for multiple tests.

For *R. leschenaulti*, the mean number of alleles per locus ranged from 12.1 (Cheranmahadevi) to 15.5 (Haikou) while the observed heterozygosity ranged from 0.85 (Cheranmahadevi) to 0.95 (Maoming). Allelic richness (R_S_) ranged from 11.0 (Wuming) to 12.2 (Menglun) (Table 2). For *Cynopterus sphinx*, the number of alleles per locus ranged from 7.1 (Mandiyoor) to 12.6 (Xishuangbanna) while the observed heterozygosity (Ho) ranged from 0.73 (Beihai) to 0.84 (Zhongshan). The allelic richness (A_R_) ranged from 6.5 (Mandiyoor, India) to 8.9 (Xishuangbanna) (Table 2). Total probability of identity (PID) values for each marker set were calculated to be 5.5×10^−18^ for *Rousettus* and 1.5×10^−4^ for *Cynopterus*, indicating sufficient power for our analyses (Table S1).

**Table 2:** Genetic variability based on cytochrome *b* and microsatellites recorded for a) *Rousettus leschenaulti* and b) *Cynopterus sphinx.*

			cytochrome *b*						microsatellites				
	Locality	n	h	H	S	K	π	n	MNA	He	Ho	R_S_	FIS
a)	Cheranmahadevi	6	5	0.93	23	1.00	0.0086	20	12.1	0.87	0.85	11.3	0.055
	Menglun	5	5	1.00	22	1.00	0.0086	25	14.0	0.88	0.87	12.2	0.037
	Wuming	6	6	1.00	21	1.00	0.0069	32	14.2	0.87	0.94	11.0	−0.062
	Maoming	6	5	0.93	16	0.93	0.0063	39	14.8	0.87	0.95	11.3	−0.070
	Haikou	6	6	1.00	19	1.00	0.0070	41	15.5	0.88	0.86	11.5	0.043
	Total	29	27	0.99	52	0.99	0.0073	157	14.1	0.87	0.89	11.4	−0.007
b)	Mandiyoor	8	7	0.96	20	2.68	0.0025	19	7.1	0.78	0.82	6.5	−0.020
	Xishuangbanna	8	7	0.96	25	6.75	0.0063	31	12.6	0.83	0.82	8.9	0.027
	Beihai	7	6	0.95	24	8.19	0.0076	22	11.0	0.82	0.73	8.8	0.139
	Jiangmen	8	4	0.82	6	2.50	0.0023	14	9.3	0.81	0.74	8.2	0.116
	Guangzhou	8	4	0.64	9	2.25	0.0021	64	11.8	0.82	0.77	7.8	0.069
	Zhongshan	7	5	0.86	10	4.00	0.0037	1	7.9	0.79	0.84	7.6	−0.022
	Haikou	6	5	0.93	10	4.40	0.0041	36	11.6	0.81	0.74	8.1	0.105
	Pu Huong	7	7	1.00	26	10.0	0.0093	14	8.4	0.81	0.78	8.2	0.085
	Total	59	34	0.96	71	10.5	0.0098	218	9.5	0.81	0.78	8.0	0.062

n =  sample size, h =  no. of haplotypes, H =  haplotype diversity, S =  no. polymorphic sites, K =  no. pairwise nucleotide differences, π =  nucleotide diversity, MNA  =  Mean no. alleles per locus, Ho =  observed heterozygosity, He =  expected heterozygosity, R_S_ =  allelic richness, FIS =  inbreeding coefficient.

### Genetic population structure

Levels of population genetic differentiation appeared to differ between the two species. Global F_ST_ for *C. sphinx* was considerablly greater (F_ST_ = 0.0235, P<0.001) than that recorded for *R. leschenaulti* (F_ST_ = 0.0067, non-significant). Pairwise F_ST_ values among *C. sphinx* samples ranged from 0.0090 (non-significant) for Pu Huong vs. Jiangmen to 0.0801 (P<0.001) for Jiangmen vs. Mandiyoor with 22 out of 28 pairwise values found to be significant (Table S2). Pairwise values among *R. leschenaulti* samples were significantly different in just three out of ten comparisons (all involving comparisons with Cheranmahadevi and Haikou) (Table S3). Although *C. sphinx* was sampled at more localities than was *R. leschenaulti*, thus precluding a meaningful comparison of their overall level of differentiation, the above species trend was also evident for those comparisons that were exactly or approximately geographically matched.

Global R_ST_ was not significantly different from global _p_R_ST_ (P<0.05) in either species indicating that stepwise mutation had not contributed to observed differentiation and that F_ST_ is a more suitable estimator in this study. Hierarchical AMOVAs based on F_ST_ revealed that more genetic variance was partitioned among individuals within populations than among populations in *C. sphinx* (96.62% versus 3.38% respectively) and *R. leschenaulti* (99.48% versus 0.52% respectively). Removing the populations from India did not significantly alter the result. *C. sphinx* was also found to exhibit a significant pattern of isolation by distance based on pairwise F_ST_ (*R* ^2^ = 0.778; P<0.01). This analysis was not undertaken for *R. leschenaulti* due to the smaller number of pairwise comparisons.

**Figure 2:**
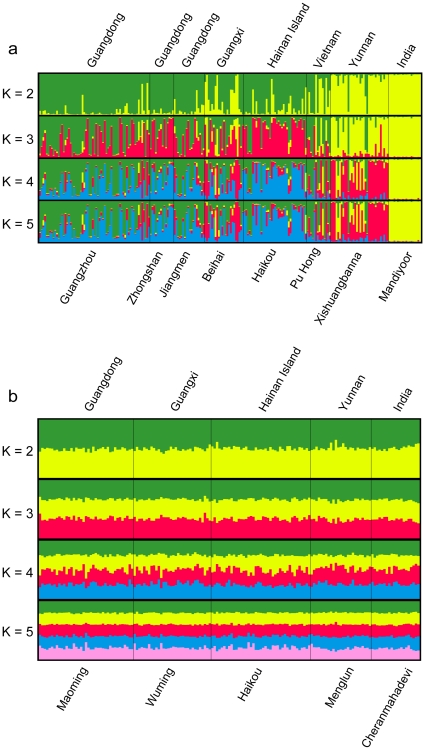
Clustering analysis for *Cynopterus sphinx* and *Rousettus leschenaulti.* Clustering analysis for a) eight *Cynopterus sphinx* populations and b) five *Rousettus leschenaulti* populations. The different colors represent the proportional membership of individuals from each locality to a given cluster, undertaken for increasing numbers of clusters (K) using STRUCTURE.

In agreement with F-statistics, Bayesian clustering analyses also revealed differences between the two taxa (Figure 2). For *C. sphinx*, K = 4 showed highest probability, however, forcing values of K from 2 to 8 recovered hierarchical population structure. At K = 2, Mandiyoor in India and Xishuangbana in SW China formed a single cluster with all other populations forming a second cluster, though partial admixture was observed. At K = 3, Mandiyoor and Xishuangbana remained as a single cluster, while the five populations from China showed partial membership to the remaining two clusters, one of which being dominated by the colony from Haikou (Hainan Island). Pu Huong appears to share mixed ancestry from all the sampled populations. At K = 4 the Mandiyoor and Xishuangbana populations split with Xishuangbana largely forming a new cluster, although some admixture is seen, while the populations from China and Pu Huong show very little structure and show partial membership to all the clusters apart from the one for Mandiyoor. Clustering analyses revealed no substructure in the *R. leschenaulti* samples, where the most probable number of clusters was one.

### MtDNA sequence analysis

We sequenced 1100 bp of cytochrome *b* in 29 individuals of *R. leschenaulti* and recorded 27 haplotypes and 52 polymorphic sites (GenBank accessions FJ489931 - FJ489957). For *C. sphinx* we sequenced 1075 bp of cytochrome *b* in 59 individuals and found 34 haplotypes with 71 polymorphic sites (GenBank accessions FJ489958 - FJ489992). No insertions or deletions were observed in either species. For each sample, the number of haplotypes, haplotype diversity, the number of polymorphic sites, average number of differences and nucleotide diversity are given in Table 1.

**Figure 3:**
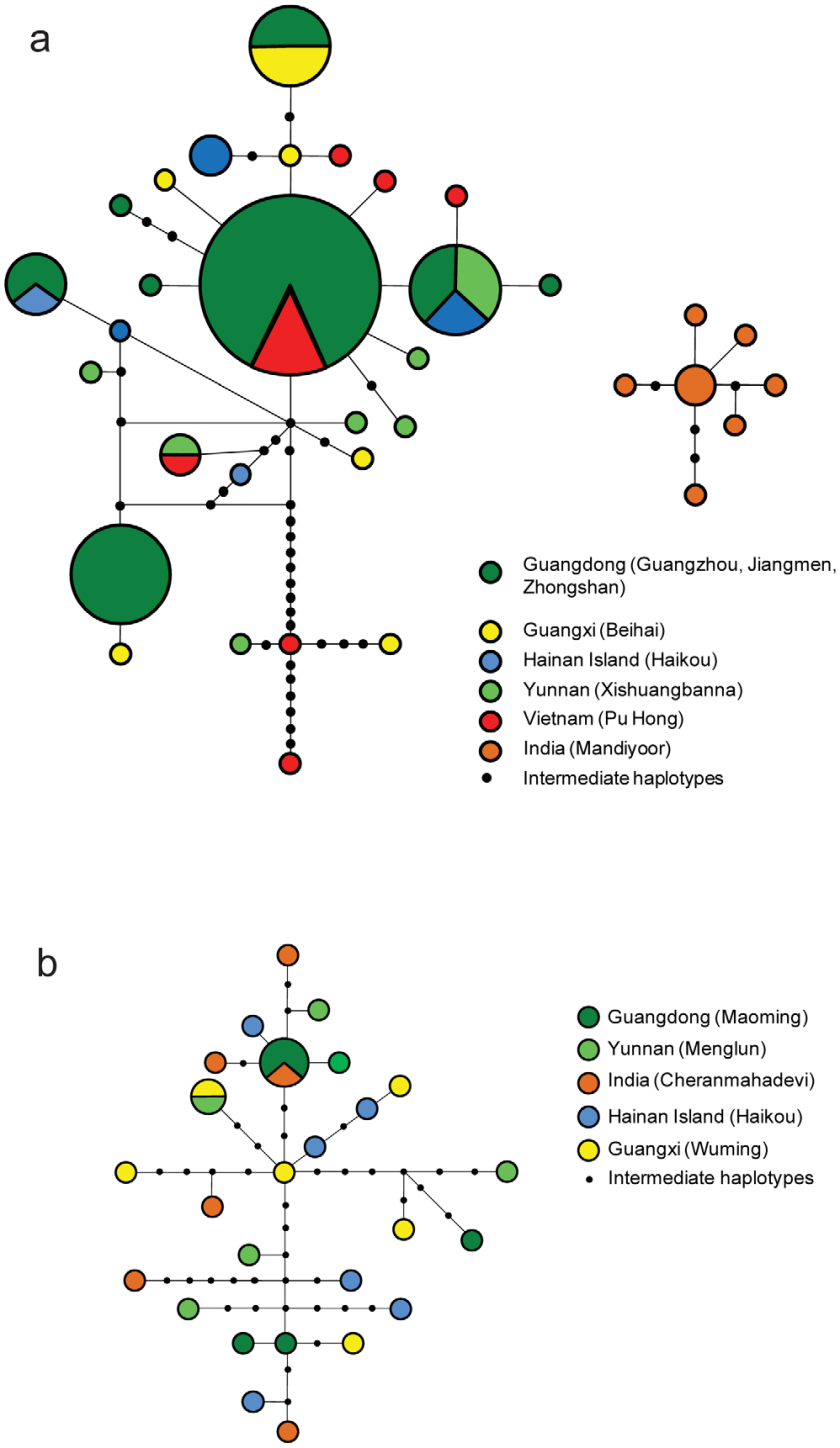
Parsimony haplotype networks for *Cynopterus sphinx* and *Rousettus leschenaulti.* Parsimony haplotype networks for a) *Cynopterus sphinx* and b) *Rousettus leschenaulti*. Haplotypes are colour coded based on sampling locality, as follows: Guangdong  =  dark green, Yunnan  =  light green, Hainan  =  blue, Guangxi  =  yellow, Vietnam  =  red, India  =  pink. Circles are sized in proportion to the number of individuals with that haplotype.

Parsimony-based haplotype networks revealed differences between the two taxa (Figure 3). For *R. leschenaulti*, haplotypes formed a single network at a 95% parsimony threshold, and were scattered with respect to locality. No evidence of any structure was present. The most common haplotype was geographically widespread, recorded in both Maoming and Cheranmahadevi. In *C. sphinx*, a parsimony threshold of 95% yielded two subnetworks, one comprising only haplotypes from Mandiyoor and the other comprising the remaining haplotypes. The most common haplotype was found mainly in Guangzhou, Zhongshan and Jiangmen but was also recorded in Pu Huong. Based on its interior position and high level of connectedness, it is likely to be ancestral. The lack of monophyly seen in haplotypes from Haikou, Beihai and Xishuangbanna points to past mixing among these localities.

No genetic differentiation was detected based on mtDNA data (global exact test, P>0.05) for *R. leschenaulti* populations. In *C. sphinx*, significant genetic differentiation was detected (global exact test, P<0.05) with pairwise values indicating that this is caused by the population sampled at Mandiyoor which was consistently differentiated from all other colonies sampled in China and Vietnam (P<0.05). Apart from the tests including Mandiyoor, no significant differentiation was detected between any other pairwise values.

AMOVAs carried out for *R. leschenaulti* showed no significant genetic variance among populations, with or without Cheranmahadevi, with 3.90% of the variance explained by among-population variation, and 96.10% by within-population variation (Table S4). AMOVAs for *C. sphinx* revealed that significant genetic variance was attributable to the two hierarchical levels examined (among and within populations). When all eight populations were included in the analysis (AMOVA I), 55.11% of the total genetic variance was explained by among population differences, and 44.89% by within population differences, however, when Mandiyoor was excluded (AMOVA II), these values were reduced to 3.04% and 96.96%, respectively (Table S4).

Mismatch distribution analysis for *R. leschenaulti* failed to reject the model of population expansion (P_SSD_>0.05 and raggedness index P_R_>0.05) with an estimated timing of expansion occurring around 200 000 years BP (95% CI: 130 000–250 000 years BP). In *C. sphinx*, separate mismatch distribution analyses undertaken for all localities also failed to reject the expansion model (P_SSD_>0.05 and raggedness index P_R_>0.05), with an estimated timing of expansion of around 107 000 years BP (95% CI: 16 000–560 000 years BP).

## Discussion

In this study we applied mtDNA and microsatellite analyses to characterize the phylogeographic histories of co-distributed populations of *Rousettus leschenaulti* and *Cynopterus sphinx.* We show that in spite of their similar ranges, these two fruit bat species exhibit highly contrasting patterns of genetic structure, and that these differences are more likely to reflect differences in vagility rather than demographic history.

Populations of *R. leschenaulti* were characterized by a lack of genetic structure at both classes of molecular marker. Our MSN network showed that haplotypes were not geographically structured while tests of AMOVA indicated that 96% of the genetic variance was due to within-population variation. Microsatellite data also revealed complete mixing of localities based on clustering analyses of genotypes which was further supported by limited evidence of genetic differentiation from F_ST_ statistics.

In contrast, haplotype data for *C. sphinx* showed a lack of genetic structure among all populations sampled across China and Vietnam but significant genetic differentiation between each of these populations and that from Mandiyoor (India). These results were also reflected in the distribution of haplotypes in the MSN network, in which the Indian haplotypes formed a monophyletic sub-network. Greater substructure was detected from microsatellite data, with significant genetic differentiation seen among most pairs of populations, including 11 pairwise comparisons within China. Bayesian clustering of genotypes also revealed clear subdivision. Specifically, we found that samples from Yunnan in Southwest China showed greater similarities with samples from India than those from elsewhere in China and Vietnam. Sampling of additional populations in the adjoining regions would help to verify these results and delimit the geographical extent of these haplotype affiliations.

A lack of genetic structure can reflect either gene flow or a recent expansion. We tested whether broad differences between our two focal taxa could be explained by differences in their demographic history. Although we detected expansions in both taxa, we estimated that the timing of population growth in the more genetically homogenous *R. leschenaulti* occurred around 200,000 years BP, which was earlier than the date of inferred growth for *C. sphinx* (107,000 years BP). Our results therefore indicate that the lack of genetic structure in *R. leschenaulti* is unlikely to reflect a recent expansion but instead results from a high level of gene flow among localities, both contemporary and in the past. In *C. sphinx*, however, the comparatively higher level of structure appears to have resulted from restricted gene flow since a more recent expansion.

Although our results are based on few and unevenly distributed populations, and so should be treated cautiously, the observed differences in genetic structure observed between *R. leschenaulti* and *C. sphinx* are consistent with our predictions based on their different roosting ecology. Studies in India and China have shown that *R. leschenaulti* migrates seasonally, with maternity colonies reaching numbers of up to 20,000 individuals during the summer and decreasing to only a few individuals each winter [33], [34]. Therefore, depending on whether mating occurs before or during migration this could provide further opportunities to promote gene flow [but see: [35]]. In contrast, *C. sphinx* forms smaller colonies, often in palm trees, and typically comprising a single male with multiple females [36]. A previous study of this taxon at a landscape scale revealed a high variance in male reproductive success yet only moderate genetic differentiation among groups [37]. In our study, mist netting of *C. sphinx* at foraging sites rather than at roosts was undertaken to reduce the potential over-representation of relatives in our samples; however, we cannot rule the possibility that this has contributed to observed subdivision.

In addition to the overall trend of restricted gene flow, our results of *C. sphinx* from Yunnan also revealed a marked discontinuity in allele frequencies within China and Vietnam, pointing to a phylogeographic signature. In particular, we found that bats sampled from Yunnan clustered with more geographically distant individuals from India rather than with other bats in China and Vietnam. In recent years, deep genetic subdivision in Southwest China, with associated suggestions of multiple glacial refugia and contact zones in this region, have been reported for several taxa, including the greater horseshoe bat [38] and the ring-necked pheasant [39]. Although the sampling regime in this study cannot resolve or explain such subdivisions, our results do hint at the possibility of multiple refugia. Our network analyses of the cytochrome *b* sequence data based on a parsimony threshold of 95% confirmed the monophyly of the Indian samples previously described [40]. This distinction was also reflected in the contrasting degree of population subdivision recorded when India was either included (Table S4, 55.11%, AMOVA I) or excluded (among population, 3.04%, AMOVA II) from our analyses, so supporting earlier suggestions that *C. sphinx* in India and China probably belong to different subspecies [41]. Interestingly, however, no such pattern was seen in the corresponding AMOVA results based on the microsatellites (among population variance 3.38% and 2.52% with and without the Indian sample, respectively). Given that both of the corresponding pairwise Fst values were not especially high, one possible explanation for this result is male-biased gene flow, either current or in the past. Alternatively, and arguably more reasonable given the scale involved, the low Fst between India and China could reflect microsatellite allele size homoplasy, which can sometimes result in erroneously low Fst values between geographically distant samples [e.g. [5]].

Comparative studies of co-distributed taxa provide a powerful means of disentangling the relative impact of biotic and abiotic factors on population genetic structure [e.g. [42], [43]]. A recent study of five passerine bird species from the Tibetan Plateau revealed that species-specific demographic histories depended on habitat requirements and, therefore, local distributions [44]. In contrast, the bats in our study have broadly similar habitat requirements, and thus the observed differences in genetic structure are probably more likely to be shaped by roosting and behavioural differences that hold across a wider sampling area. Our findings support a number of papers that have linked social structure to genetic differentiation [e.g. [45]], and also corroborate suggestions that colonial cave roosting bats are likely to show more dispersal and gene flow than do tree roosting species [10].

## Supporting Information

Table S1 PCR conditions and details for loci used in this study for a) *Rousettus leschenaulti* and b) *Cynopterus sphinx*.
(0.06 MB DOC)

Table S2 Pairwise ΦST (above the diagonal) and FST estimates (below the diagonal) for eight *Cynopterus sphinx* populations. Bold  =  significant differentiation at P<0.05.
(0.04 MB DOC)

Table S3 Pairwise ΦST (above diagonal) and FST estimates (below diagonal) for five *Rousettus leschenaulti* populations. Bold  =  significant differentiation at P<0.05.
(0.03 MB DOC)

Table S4 Results of AMOVA based on microsatellite and mtDNA data for a) *Cynopterus sphinx* and b) *Rousettus leschenaulti* populations. AMOVA I includes all populations, AMOVA II excludes the populations from India.
(0.03 MB DOC)
