# Supplementary material for: Contrasting Genetic Structure in Two Co-Distributed Species of Old World Fruit Bat
Source: PLoS One. 2010 Nov 10;5(11):e13903. doi: 10.1371/journal.pone.0013903 (PMC2978090; doi:10.1371/journal.pone.0013903)
Supplement: Table S1 — PCR conditions and details for loci used in this study for a) Rousettus leschenaulti and b) Cynopterus sphinx. (0.06 MB DOC) [file pone.0013903.s001.doc]

Supplementary Table 1. PCR conditions and details for loci used in this study for a) *Rousettus leschenaulti* and b) *Cynopterus sphinx*.

|  | Locus | Reference* | Tm (°C) | Allele range (bp) | PID | GenBank accession |
| --- | --- | --- | --- | --- | --- | --- |
| a) | M3-8 | A | 65 | 138-166 | 0.033 | DQ389096 |
|  | M3-6 | A | 65 | 152-202 | 0.007 | DQ389097 |
|  | M3-120 | A | 61 | 168-226 | 0.048 | DQ389099 |
|  | M3-121 | A | 65 | 160-196 | 0.019 | DQ389100 |
|  | M3-3 | A | 65 | 149-207 | 0.009 | DQ389101 |
|  | M3-1 | A | 61 | 153-211 | 0.023 | DQ389102 |
|  | B04 | B | 52 | 102-138 | 0.017 | EF531084 |
|  | D04-2 | B | 52 | 201-243 | 0.029 | EF531085 |
|  | D10-2 | B | 52 | 272-324 | 0.013 | EF531086 |
|  | D12-2 | B | 52 | 201-251 | 0.020 | EF531087 |
|  | Mean |  |  |  | 5.509x10^-18^ |  |
|  |  |  |  |  |  |  |
| b) | AF02 | C | 57 | 95-155 | 0.351 | AF289706 |
|  | AF03 | C | 57 | 92-170 | 0.391 | AF289707 |
|  | AF04 | C | 59 | 115-163 | 0.349 | AF289708 |
|  | AF05 | C | 55 | 94-206 | 0.316 | AF289709 |
|  | AF06 | C | 58 | 111-277 | 0.296 | AF289710 |
|  | AF07 | C | 57 | 211-285 | 0.286 | AF289711 |
|  | AF08 | C | 57 | 114- 218 | 0.316 | AF289712 |
|  | AF09 | C | 57 | 274-302 | 0.370 | AF289713 |
|  | Mean |  |  |  | 1.497 x10^-4^ |  |

*Loci are described in ref. A (Hua *et al*. 2006), ref. B (Shao *et al*. 2007) and ref. C (Storz *et al*. 2000).

Tm, annealing temperature. PID, probability of identity.
