# Supplementary material for: Contrasting Genetic Structure in Two Co-Distributed Species of Old World Fruit Bat
Source: PLoS One. 2010 Nov 10;5(11):e13903. doi: 10.1371/journal.pone.0013903 (PMC2978090; doi:10.1371/journal.pone.0013903)
Supplement: Table S2 — Pairwise ΦST (above the diagonal) and FST estimates (below the diagonal) for eight Cynopterus sphinx populations. Bold = significant differentiation at P<0.05. (0.04 MB DOC) [file pone.0013903.s002.doc]

Supplementary Table 2. Pairwise Φ_ST_ (above the diagonal) and F_ST_ estimates (below the diagonal) for eight *Cynopterus sphinx* populations. Bold = significant differentiation at *P* < 0.05

| Population | Guangzhou | Zhongshan | Jiangmen | Beihai | Haikou | Pu Huong | Xishuangbanna | Mandiyoor |
| --- | --- | --- | --- | --- | --- | --- | --- | --- |
| Guangzhou | - | 0.118 | 0.073 | 0.047 | 0.055 | 0.085 | 0.014 | **0.906** |
| Zhongshan | **0.016** | - | 0.015 | -0.002 | 0.053 | 0.075 | 0.015 | **0.879** |
| Jiangmen | **0.012** | 0.018 | - | 0.076 | 0.084 | 0.088 | -0.014 | **0.904** |
| Beihai | **0.019** | **0.024** | 0.010 | - | 0.034 | -0.034 | -0.011 | **0.809** |
| Haikou | **0.018** | 0.012 | 0.015 | **0.029** | - | 0.044 | -0.023 | **0.877** |
| Pu Huong | **0.022** | **0.032** | 0.009 | 0.019 | **0.025** | **-** | **0.027** | **0.768** |
| Xishuangbanna | **0.032** | **0.038** | **0.032** | **0.019** | **0.040** | -0.060 | - | **0.823** |
| Mandiyoor | **0.079** | **0.071** | **0.080** | **0.066** | **0.072** | **0.077** | **0.060** | - |
