# Supplementary material for: Contrasting Genetic Structure in Two Co-Distributed Species of Old World Fruit Bat
Source: PLoS One. 2010 Nov 10;5(11):e13903. doi: 10.1371/journal.pone.0013903 (PMC2978090; doi:10.1371/journal.pone.0013903)
Supplement: Table S3 — Pairwise ΦST (above diagonal) and FST estimates (below diagonal) for five Rousettus leschenaulti populations. Bold = significant differentiation at P<0.05. (0.03 MB DOC) [file pone.0013903.s003.doc]

Supplementary Table 3. Pairwise Φ_ST_ (above diagonal) and F_ST_ estimates (below diagonal) for five *Rousettus leschenaulti* populations. Bold = significant differentiation at P < 0.05.

| Population | Maoming | Wuming | Haikou | Menglun | Cheranmahadevi |
| --- | --- | --- | --- | --- | --- |
| Maoming | - | -0.007 | -0.023 | -0.068 | -0.025 |
| Wuming | -0.003 | - | 0.031 | -0.059 | -0.060 |
| Haikou | 0.006 | **0.009** | - | 0.015 | -0.077 |
| Menglun | 0.005 | 0.002 | 0.006 | - | -0.033 |
| Cheranmahadevi | **0.013** | **0.015** | 0.010 | 0.011 | - |
