# Supplementary material for: Contrasting Genetic Structure in Two Co-Distributed Species of Old World Fruit Bat
Source: PLoS One. 2010 Nov 10;5(11):e13903. doi: 10.1371/journal.pone.0013903 (PMC2978090; doi:10.1371/journal.pone.0013903)
Supplement: Table S4 — Results of AMOVA based on microsatellite and mtDNA data for a) Cynopterus sphinx and b) Rousettus leschenaulti populations. AMOVA I includes all populations, AMOVA II excludes the populations from India. (0.03 MB DOC) [file pone.0013903.s004.doc]

Supplementary Table 4. Results of AMOVA based on microsatellite and mtDNA data for a) *Cynopterus sphinx* and b) *Rousettus leschenaulti* populations. AMOVA I includes all populations, AMOVA II excludes the populations from India.

|  |  | AMOVA I | | | AMOVA II | |
| --- | --- | --- | --- | --- | --- | --- |
|  |  | microsatellite | mtDNA | | microsatellite | mtDNA |
|  | Source of variation | % of variation | | | % of variation | |
|  |  |  | | |  | |
| a) | Among populations | 03.38 |  | 55.11 | 02.52 | 03.04 |
|  | Within populations | 96.62 |  | 44.89 | 97.48 | 96.96 |
|  |  |  |  |  |  |  |
| b) | Among populations | 01.48 |  | 03.90 | 01.12 | 02.52 |
|  | Within populations | 98.52 |  | 96.10 | 98.88 | 97.48 |
